# A Multi-Sensor Fusion MAV State Estimation from Long-Range Stereo, IMU, GPS and Barometric Sensors

**DOI:** 10.3390/s17010011

**Published:** 2016-12-22

**Authors:** Yu Song, Stephen Nuske, Sebastian Scherer

**Affiliations:** 1 Robotics Institute, Carnegie Mellon University, Pittsburgh, PA 15213, USA; nuske@cmu.edu (S.N.); basti@andrew.cmu.edu (S.S.); 2 School of Electronic and Information Engineering, Beijing Jiaotong University, Beijing 100044, China

**Keywords:** multi-sensor fusion, GPS-denied state estimation, long-range stereo visual odometry, absolute and relative state measurements, stochastic cloning EKF

## Abstract

State estimation is the most critical capability for MAV (Micro-Aerial Vehicle) localization, autonomous obstacle avoidance, robust flight control and 3D environmental mapping. There are three main challenges for MAV state estimation: (1) it can deal with aggressive 6 DOF (Degree Of Freedom) motion; (2) it should be robust to intermittent GPS (Global Positioning System) (even GPS-denied) situations; (3) it should work well both for low- and high-altitude flight. In this paper, we present a state estimation technique by fusing long-range stereo visual odometry, GPS, barometric and IMU (Inertial Measurement Unit) measurements. The new estimation system has two main parts, a stochastic cloning EKF (Extended Kalman Filter) estimator that loosely fuses both absolute state measurements (GPS, barometer) and the relative state measurements (IMU, visual odometry), and is derived and discussed in detail. A long-range stereo visual odometry is proposed for high-altitude MAV odometry calculation by using both multi-view stereo triangulation and a multi-view stereo inverse depth filter. The odometry takes the EKF information (IMU integral) for robust camera pose tracking and image feature matching, and the stereo odometry output serves as the relative measurements for the update of the state estimation. Experimental results on a benchmark dataset and our real flight dataset show the effectiveness of the proposed state estimation system, especially for the aggressive, intermittent GPS and high-altitude MAV flight.

## 1. Introduction

Light weight Micro-Aerial Vehicles (MAVs) equipped with sensors can autonomously access environments that are difficult to access for ground robots. Due to this capability, MAVs have become popular in many robot missions, e.g., structure inspection, environment mapping, reconnaissance and large-scale data gathering. Compared with ground robots, there are two main challenges for MAV autonomous navigation: (1) limited payload, power and onboard computing resources, so only light-weight compact sensors (like cameras) can be integrated for MAV applications; and (2) MAVs usually move with fast and aggressive six DOF (Degrees Of Freedom) motions. Accordingly, their state estimation, environment perception and obstacle avoidance are more difficult than ground robots.

Robust, accurate and smooth high-rate state estimation is the most critical capability to realize truly autonomous flight of MAVs. The state estimator reports the six DOF MAV pose and the velocity, so the output of the estimator serves as the input for environment mapping, motion planning and trajectory-following control. GPS (Global Positioning System) combined with the IMU (Inertial Measurement Unit) state estimation technique has been widely utilized for providing MAV high-rate state information. Applications of low-rate GPS are limited to open environments, and also, GPS cannot provide accurate positioning information for MAV, especially in terms of altitude. As a complimentary sensor for GPS, the IMU measures the tri-axis accelerations and rotation rates in the IMU body frame, and the velocity and orientation are calculated as the integral of accelerations and rotation rates over time. For low-cost commercial IMUs, the inertia integral will drift very fast without global rectification information. As a result, the integration of additional sensing is a possible way to further improve state estimation redundancy, accuracy and robustness.

Because of the low cost, low energy consumption and satisfactory accuracy, camera-based Visual Odometry (VO) is an ideal choice for providing additional measurements. Stereo visual sensors reconstruct the environment features with the metric scale from the stereo baseline, so stereo-based VO easily generates six DOF pose measurements. The performance of stereo VO highly depends on the ratio between the stereo baseline and environmental depth, namely the baseline-depth ratio. The depth standard deviation from stereo is proportional to the quadratic of depth; thus, stereo VO is limited to a short range. As with the results reported in reference [1], at stereo disparities lower than 10 pixels, the depth triangulation from a single stereo rig tends to follow a non-Gaussian curve with a long tail. For cases with a large baseline-depth ratio (e.g., MAV high-altitude flights), stereo almost degenerates to a monocular system, thus losing the capability of pose measurements.

In this paper, we present a state estimation system that utilizes long-range stereo odometry that can degrade to a monocular system at high altitude and integrates GPS, barometric and IMU measurements. The estimation system has two main parts: an EKF (Extended Kalman Filter) estimator that loosely fuses both absolute state measurements (GPS, barometer) and the relative state measurements (IMU, VO) is derived and discussed in detail; a long-range stereo VO is designed both for low- and high-altitude odometry calculation. The odometry takes the EKF prediction information for robust camera pose tracking and feature matching, and the stereo VO outputs serve as the relative measurements for the update of the EKF state. There are three main highlights for the system:

(1) The state estimation system utilizes both absolute state measurement sensors (GPS, barometer), the relative six DOF pose state measurement provided by VO. To deal with both absolute and relative state measurements effectively, we derive a new stochastic cloning EKF state estimator to generate accurate and smooth state estimation both for GPS-available and GPS-denied environments.
(2) We developed a robust long-range stereo VO that works well both for low- and high-altitude cases. At low altitude, the VO utilizes stereo images; that means the features are directly triangulated by stereo pairs with a fixed static stereo baseline. At high altitude, the ratio between the scene depth and stereo baseline becomes large, and the stereo pair almost degenerates to a monocular system. In this situation, the additional stereo observations over time are fused by both multi-stereo triangulation and a multi-view stereo inverse depth filter for long-range feature depth generation.
(3) The EKF estimator and long-range VO coordinate to improve the robustness of the method. The IMU integral prediction information from the EKF estimator is used both for guiding image-feature matching and long-range VO optimization. Additionally, the VO is utilized as the relative measurement for the update of the EKF state.

The rest of the paper is organized as follows: Section 2 reviews the related work. Section 3 presents the proposed long-range stereo VO. In Section 4, a new EKF state estimator that combines both absolute and relative state measurements (GPS, barometer, IMU and long-range stereo VO) is derived and discussed. Finally, the experimental results will be reported and analyzed, followed by conclusions in Section 5.

## 2. Related Work

Since the VO concept was first derived, many VO algorithms have been proposed. Monocular VOs were developed on the basis of the “structure from motion” idea in computer vision. The first monocular VO uses a fundamental matrix between the two images to recover the camera motion [2]. As a milestone for modern VO or vSLAM (visual Simultaneous Localization And Mapping), PTAMwas proposed by Klein et al. in 2009 [3]. PTAM utilizes two threads for odometry: one is for sparse-features local mapping optimization, and the other one is for online camera pose tracking on the built local map. The original PTAM can only be used for small environments, and its modified versions have been applied for MAV state estimation with a down-looking camera system [4]. With a similar idea to PTAM, some other monocular VO or SLAM algorithms were proposed. For example, SVO [5], which was proposed by Foster et al. in 2014, utilizes image patches for camera direct tracking, because the features are only detected for selected key-frames, and also, no descriptors are extracted; therefore, SVO is faster than PTAM. At the same time, SVO was limited for high-rate cameras since direct tracking is employed. SDVO [6] and LSD-SLAM [7] are the first semi-dense monocular VO and SLAM algorithms. The idea for LSD-SLAM is also from the PTAM framework, but using high-gradient pixels for camera pose tracking (frame to key-frame tracking) and using the pose tracking result to refine the semi-dense depth of key-frames. As a state-of-the-art sparse feature VO or SLAM approach, ORBSLAM [8] has an additional thread for loop-closure detection and global batch optimization. For MAV state estimation using a monocular camera, there are three main limitations: (1) it is difficult for initial map generation since a single image cannot provide depth information; (2) the translation is up to scale, and the scale easily drifts over time; therefore, it usually should be combined with other sensors like an IMU or laser to recover the absolute scale; and (3) the monocular VO is not robust enough for MAVs’ fast motions, unless high-rate cameras are utilized.

As a result, the stereo and RGBD sensors are more suitable for MAV application. Due to the limited range for depth perception (<4 m), most RGBD VOs are designed for indoor environments. As the first direct VO technique, DVO [9] utilizes the photometric projection consistency to track the current camera pose with respect to (w.r.t.) the last pose by using both depth and color images. Since RGBD cameras have the ability of dense depth perception, some point-cloud matching techniques have also been used [10]. A monocular-RGBD combined approach was proposed in 2015 [11], in which the key idea is to triangulate features that lack effective depth perception (>4 m). Libviso2 [12] and FOVIS [13] are the two well-known algorithms for stereo VO. Both algorithms utilize “reprojection error minimization” to calculate six DOF odometry. In stereo PTAM [14], the correspondence between the map points and current features is matched with a constant velocity motion model. Stereo version ORBSLAM follows the same idea as the monocular ORBSLAM and uses the static stereo baseline to reconstruct the map points both for map initialization and odometry calculation. As a result, the stereo version ORBSLAM is more robust than the monocular one. Stereo LSDSLAM is derived from monocular LSDSLAM, and the main difference lies in the direct utilization of dense stereo disparity for key-frame depth generation [15]. The SOFTalgorithm [16] was designed on the basis of Libviso2. The main contribution lies in the decoupling of the rotation and translation calculations: the five-point algorithm is utilized to calculate the rotation motion first, then with the reprojection error minimization for translation optimization. Recently, a long-range stereo VO algorithm was proposed with a 0.006 baseline-depth ratio. The stereo baseline is 2 m, and the maximum altitude is 120 m for a fixed-wing MAV. In this approach, an initial map is estimated with a monocular technique using only stereo left images, and then, the rejection errors of the reconstructed map points on the stereo right images are considered to recover scale. In this way, even the stereo baseline is very weak compared with environmental depth; it still provides useful metric scale information for batch optimization. Furthermore, the multi-view stereo bundle adjustment technique is used by taking the stereo right images as additional views for map point triangulation. While the algorithm cannot operate online, as reported in the experimental results, the long-range VO runs 1.35 Hz on an offline desktop PC [1].

For the fusion state estimation, the approaches can be classified into loosely coupled and tightly coupled in terms of sensor information utilization. For loosely-coupled algorithms, various sensors generate state measurements independently, and fuse the state measurements using either filters (EKF, UKF, PF) or smoothers (G2O [17], iSAM [18]). For loosely-coupled methods, the state estimation rate is equal to the highest state measurement rate. As a result, loosely-coupled approaches are suitable for high-rate requirements, e.g., for MAV control. Furthermore, loosely-coupled methods are more robust to sensor failures. Weiss et al. [4,19] proposed a multi-sensor fusing approach with IMU and monocular SLAM for MAV. The technique utilizes modified PTAM to provide an absolute pose measurement, so other absolute sensors are limited for integration. Furthermore, the system state will slowly drift due to the SLAM error accumulation over time. A UKF loosely-coupled state estimator was proposed for fusing stereo VO, GPS, IMU and barometer [20]. The estimator utilizes the unscented transform to calculate the transition probability densities. Accordingly, no Jacobians are required, and the transition probability densities can be computed up to the third non-linearity. Because the state vector has 21 elements, 43 sigma points will be propagated both for prediction and updates. The computational cost is much higher than an EKF. In reference [21], a stochastic cloning Kalman filter was proposed for fusing relative state measurements by augmenting state with the delayed pose. With this idea, the IMU and VO are fused in an EKF framework [22]. For tightly-coupled approaches, the sensors do not report state estimates on their own. Instead, all of the sensing information is combined to calculate a final output. It has been proven that tightly-coupled approaches outperform loosely-coupled algorithms in terms of accuracy. The EKFSLAM and MSCKFare two filter-based tightly-coupled VO/IMU fusion approaches [23]. In EKF-SLAM, the positions of visual landmarks in a sliding window are integrated. The IMU integral is used for pose and velocity prediction, and the sliding window SLAM system will be updated by visual landmark re-observations. In MSCKF, the system state is augmented by *N* delayed IMU poses; the delayed IMU poses are connected by visual landmarks. This is similar to the local bundle adjustment without iterated relinearization. Leutenegger et al. proposed a key-frame-based visual-inertial odometry with local bundle adjustment, with special focus on the marginalization of measurement terms outside the bundle adjustment window [24]. For smoother-based visual-inertia odometry, the IMU integral has to be recalculated because of the change of the linearization point in the batch optimization iteration steps. To reduce the re-integral computational cost, an IMU pre-integral approach in the IMU body frame has been discussed in reference [25].

## 3. Long-Range Stereo Odometry

The sparse features-based stereo VO algorithms are popular for robotics navigation applications. A key aspect of the stereo VO is to minimize a nonlinear error cost function by projecting the local map 3D points or 3D points generated from the reference stereo frame to the current stereo image pair. Current stereo VO algorithms have two main limits for MAV applications: (1) a lack of robustness in fast motion, especially for rotation; and (2) no correct estimation at long-ranges. In this section, we address the two main limitations of the stereo VO implementation to make stereo VO robust for MAV long-range high-altitude applications.

### 3.1. Long-Range Stereo Odometry Pipeline

Stereo depth reconstruction with a fixed static baseline is limited to a short range. For static stereo triangulation, the feature depth *z* is associated with the stereo matching disparity *d* as: $z=f_{x}\frac{B}{d}$ (where $f_{x}$ is the focus length in pixels and *B* is the length of static stereo baseline in meters). Suppose the stereo matching disparity has variance $\sigma_{d}^{2}$; the triangulated depth variance $\sigma_{z}^{2}$ by stereo is as Equation (1). It is clear that the stereo depth standard deviation $\sigma_{z}$ is proportional to a quadratic of depth *z*. The depth error increases very quickly for the small disparity, long-range stereo measurements and, thus, cannot be utilized for VO optimization.>

(1) $$\sigma_{z}^{2}={\left(\frac{\partial z}{\partial d}\right)}^{2}\sigma_{d}^{2}=\frac{f_{x}^{2}B^{2}}{d^{4}}\sigma_{d}^{2}=\frac{z^{4}}{f_{x}^{2}B^{2}}\sigma_{d}^{2}$$

**Figure 1:**
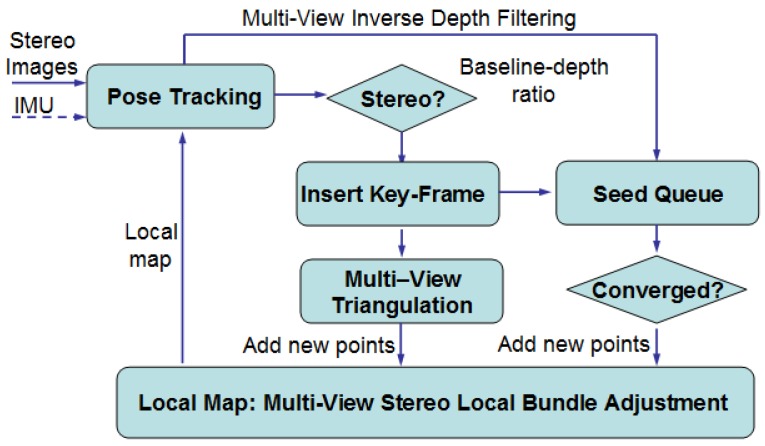
Long-range visual odometry pipeline.

**Figure 2:**
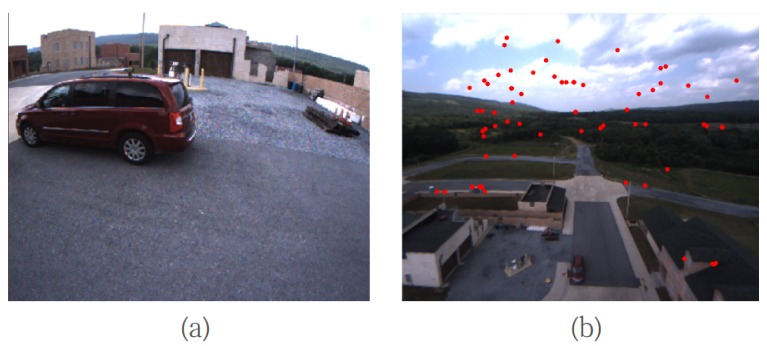
Short-range and long-range environments. (**a**) Short-range environment. In this situation, most of the features are directly triangulated by static stereo baseline. (**b**) The long-range environment can also detect some large disparities of “short-rang” features (red dot) due to incorrect matching for the repetitive texture area. For this case, most of the "short-range” features are outliers; thus, they cannot be directly triangulated by static stereo baseline.

Long-range stereo depth error (bias) can be effectively reduced by introducing additional stereo observation over time, namely multi-view stereo with a dynamic pseudo baseline. The pseudo baseline between the stereo frames can be used for the triangulation of the long-range stereo points. The fixed stereo baseline can provide an absolute scale constraint. Based on this idea, we developed a sparse feature-based stereo VO both for short- and long-range cases. The pipeline of the proposed long-range stereo VO is shown in Figure 1. It is a key-frame-based VO technique. The local map consists of a set of 3D sparse map points that is generated by selected key-frames. Furthermore, IMU information is integrated to further improve the robustness for aggressive camera motion and repetitive texture environments. Based on the current stereo baseline-depth ratio, the VO system switches both key-frame and new map point generation strategies between stereo and monocular modes:

(1) For a short range (e.g., MAV low-altitude flight, as shown in Figure 2a, the VO works with a stereo mode. For each new selected key-frame, most of the new features are directly triangulated by the stereo camera pair with the static stereo baseline. For some long-range points, they are triangulated using both the pseudo-baseline formed by the key-frame’s poses and the static stereo baseline. In stereo mode, the environment structure is close to the camera; the image context easily changes especially for camera rotation. Therefore, the key-frames and its features are inserted into the local map relatively densely.
(2) For a long range (e.g., high-altitude flight, as shown in Figure 2b, the VO switches to monocular mode. The key-frames are inserted sparsely to provide enough relative motion between the key-frames for long-range triangulation. When VO is in a long-range mode, no features will be directly triangulated by static stereo. Because most of the “short-range points” will be outliers due to an incorrect matching from a low or repetitive texture area, such as sky, cloud and trees, instead, the new features will first be triangulated using both a dynamic pseudo baseline and a static stereo baseline. For the new features that cannot be triangulated by the pseudo baseline, we insert them into a “candidate queue”. The feature depth will be iteratively refined by subsequently tracking stereo information with a multi-view inverse depth filter. If the inverse depth converges, the candidate feature will be added into the map and then used for camera pose tracking.

### 3.2. Long-Range Point Generation Using Multi-View Stereo Triangulation

The most critical aspect for long-range stereo is feature depth generation. For each new key-frame, its features can be classified into three groups:

(1) the features have been matched with the map.
(2) new features with an effective stereo depth (i.e, short-range points, with enough stereo disparity).
(3) new features with small disparities (long-range points).

**Figure 3:**
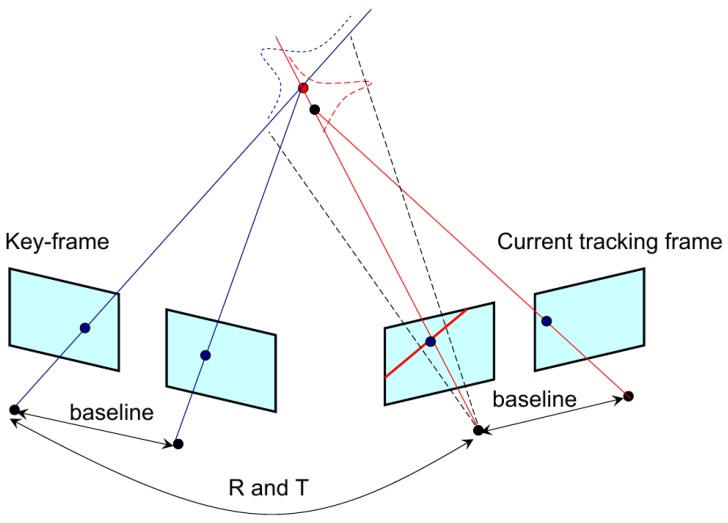
Multi-view observations by stereo. Between the two frames, the camera motions *R* and *T* provide a dynamic pseudo baseline, and for each stereo frame, the feature position is constrained by the static stereo baseline.

The new long-range points without depth will first be triangulated using both the pseudo-baseline and the static stereo baseline from multi-view stereo measurements. The pseudo baseline is formed by the “relative pose” between the neighboring key-frames. As shown in Figure 3, the current left image feature is searched in the previous key-frame’s left image feature set on the basis of an epipolar constraint, and for each key-frame, the matched feature pairs also have their own corresponding features in the right image. To make the matching more robust, the epipolar constraint between right image features is also checked. As a result, for each new map point, four matched features can be obtained between two key-frames, and the map point is triangulated as the intersection point of the four rays in the sense of least-squares.

### 3.3. Long-Range Point Generation by Multi-View Stereo Inverse Depth Filtering

**Figure 4:**
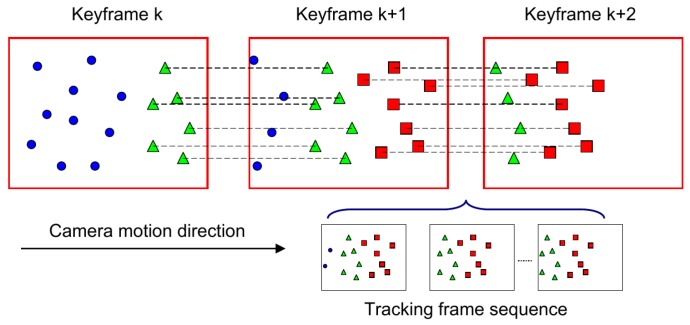
An example for the camera exploration mode. For the *k*-th key-frame; the blue points indicate the “old” features that have been matched with the map; and green triangles are the new features that await triangulation. For the (*k* + 1)-th key-frame, green triangle features can be triangulated, and some new features (red rectangle) wait for the next key-frame for triangulation. Between the (*k* + 1)-th key-frame and the (*k* + 2)-th key-frame, there is a set of tracking frames that also can provide useful measurements for the new features (red rectangle); we integrate all of the multi-view observations for the new feature using an inverse depth filter.

The inter-key-frames’ triangulation is a kind of delayed depth generation approach because only features that can be viewed by at least two key-frames can be triangulated. For the exploration mode (e.g., the stereo moves forward), there are some new features that belong to the current key-frame itself; thus, they cannot be triangulated in time. An illustrative example is shown in Figure 4. To also apply these kinds of new features for subsequent camera pose tracking, we designed an inverse depth filter for each new candidate. For stereo, the inverse depth $\rho =\frac{1}{z}=\frac{d}{f_{x}B}$ is proportional to disparity *d*; as a result, the inverse depth uncertainty is easily modeled by a Gaussian distribution:

(2) $$\sigma_{\rho }^{2}=\frac{1}{f_{x}^{2}B^{2}}\sigma_{d}^{2}$$

For each long-range candidate feature that belongs to the new inserted key-frame, its initial inverse depth prior is directly obtained from noisy static stereo depth triangulation, denoted as $N(\rho_{0},\frac{1}{f_{x}^{2}B^{2}}\sigma_{d}^{2})$. During the subsequent pose tracking, each new tracking frame is utilized to filter the initial distribution $N(\rho_{0},\frac{1}{f_{x}^{2}B^{2}}\sigma_{d}^{2})$, and the new feature candidate will be added to the map until its inverse depth variance is smaller than a given threshold. Ideally, for each new tracking frame, we can obtain two new observations for the candidate feature: (1) the inverse depth observation distribution for the candidate is calculated from the tracking frame static stereo matching; and (2) the inverse depth observation distribution can also be obtained by the dynamic pseudo baseline formed by the motion between the current tracking frame and its reference key-frame. Therefore, the filtered inverse depth distribution can be updated by the two new observations.

Denote as the 3D coordinate of a candidate feature with $z_{0}=1$ as $P_{0}={\left(x_{0},y_{0},1\right)}^{T}$ in the key-frame coordinate and its corresponding matching point in the current tracking frame with $z_{1}=1$ is $P_{1}={\left(x_{1},y_{1},1\right)}^{T}$. The motion from the key-frame to the current tracking frame is $R_{10}$, $t_{10}={\left(t_{x},t_{y},t_{z}\right)}^{T}$, so the relationship of the two points is:

(3) $$\frac{1}{\rho_{1}}P_{1}=\frac{1}{\rho_{0}}R_{10}P_{0}+t_{10}$$

where $\rho_{1}$ and $\rho_{0}$ represent the inverse depth measurements in the current tracking frame and key-frame, respectively.

For the current tracking frame, we observe the inverse depth stereo $\rho_{1}$ with its variance $\frac{1}{f_{x}^{2}B^{2}}\sigma_{d}^{2}$. Therefore, the new measured inverse depth and its variance in the key-frame coordinate are calculated by projecting the new measurement $N(\rho_{1},\frac{1}{f_{x}^{2}B^{2}}\sigma_{d}^{2})$ to the key-frame coordinate based on the last row of Equation (3):

(4) $$\begin{array}{c} \rho_{0}^{s}=\frac{\frac{1}{\rho_{1}}-t_{z}}{R_{10}\left(3\right)P_{0}}\\ \sigma_{\rho_{0}^{s}}^{2}={\left(\frac{\rho_{0}^{s}}{\rho_{1}}\right)}^{4}{\left(\frac{1}{R_{10}\left(3\right)P_{0}f_{x}B}\right)}^{2}\sigma_{d}^{2} \end{array}$$

where $R_{10}\left(3\right)$ represents the third row of rotation matrix $R_{10}$ and $\sigma_{d}^{2}$ is the new stereo disparity variance in the current tracking frame (we set $\sigma_{d}^{2}=1$).

The inverse depth triangulation distribution using the motion from the key-frame to the current tracking frame is also derived from Equation (3) (with the first row and the last row). We have:

(5) $$\begin{array}{c} \rho_{0}^{e}=\frac{R_{10}\left(1\right)P_{0}-R_{10}\left(3\right)P_{0}x_{1}}{t_{z}x_{1}-t_{x}}\\ \sigma_{\rho_{0}^{e}}^{2}={\left(\frac{R_{10}\left(3\right)P_{0}t_{x}-R_{10}\left(1\right)P_{0}t_{z}}{{\left(t_{z}x_{1}-t_{x}\right)}^{2}f_{x}}\right)}^{2}\sigma_{u1}^{2} \end{array}$$

where $R_{10}\left(1\right)$ represents the first row of rotation matrix $R_{10}$ and $\sigma_{u1}^{2}$ describes the matching error variance along the epipolar line in the current tracking frame; we set $\sigma_{u1}^{2}=4$ in our experiments.

To remove the outlier inverse depth measurements, both of the two new inverse depth hypotheses are further tested with prior $N(\rho_{0},\sigma_{\rho_{0}}^{2})$ using $\chi^{2}$ compatibility testing at 0.95. After passing the test, the posterior of the inverse depth distribution for the candidate feature is updated by multiplying the prior with the new measurements $N(\rho_{0}^{s},\sigma_{\rho_{0}^{s}}^{2})$ and $N(\rho_{0}^{e},\sigma_{\rho_{0}^{e}}^{2})$, that is:

(6) $$N\left(\rho_{0}^{+},\sigma_{\rho_{0}^{+}}^{2}\right)=N\left(\rho_{0},\sigma_{\rho_{0}}^{2}\right)N\left(\rho_{0}^{s},\sigma_{\rho_{0}^{s}}^{2}\right)N\left(\rho_{0}^{e},\sigma_{\rho_{0}^{e}}^{2}\right)$$

### 3.4. Local Bundle Adjustment for Multi-View Stereo Optimization

The long-range stereo points generated by either triangulation or inverse depth filtering may still be noisy. An effective approach to further improve the feature 3D reconstruction accuracy is multi-view stereo local Bundle Adjustment (BA). During the local BA, the re-projection errors for both left and right images are considered. If the map points are reconstructed with an incorrect scale, the re-projection error on the right images will be large. Accordingly, the “weak” static stereo baseline can provide an absolute scale constraint for local BA optimization. The Jacobian $J_{pi}$ of the rejection residual $\epsilon_{reproj}\left(i\right)$ w.r.t. the map point $P_{i}={\left(X_{i},Y_{i},Z_{i}\right)}^{T}$ is:

(7) $$J_{Pi}=\left[\begin{array}{c} \frac{\partial \epsilon_{reproj}\left(i\right)}{\partial u_{i}^{l}}\frac{\partial u_{i}^{l}}{\partial P_{i}}\\ \frac{\partial \epsilon_{reproj}\left(i\right)}{\partial v_{i}^{l}}\frac{\partial v_{i}^{l}}{\partial P_{i}}\\ \frac{\partial \epsilon_{reproj}\left(i\right)}{\partial u_{i}^{r}}\frac{\partial u_{i}^{r}}{\partial P_{i}} \end{array}\right]=-\frac{1}{Z_{c}}\left[\begin{array}{ccc} f_{x} & 0 & -f_{x}\frac{X_{c}}{Z_{c}}\\ 0 & f_{y} & -f_{y}\frac{Y_{c}}{Z_{c}}\\ f_{x} & 0 & -f_{x}\frac{X_{c}-B}{Z_{c}} \end{array}\right]R$$

where $P_{c}={\left(X_{c},Y_{c},Z_{c}\right)}^{T}$ is the map point 3D coordinate in the left camera frame system. The first two rows are the residual Jacobian w.r.t. the left image and the last row is for right image. *R* is the camera rotation matrix.

**Figure 5:**
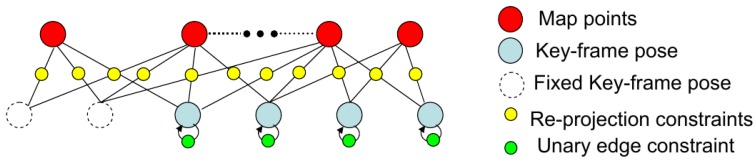
Factor graph for long-range stereo local bundle adjustment.

The factor graph for the long-range stereo is shown in Figure 5. We add a unary edge $I_{4\times 4}$ to each key-frame pose vertex. Consequently, the local BA will mainly focus on the map point optimization, and the key-frame’s pose can only be changed in a small range. The factor graph is more like a structure-only bundle adjustment since the camera pose tracking has been fused with the IMU motion information (the IMU coupled odometry will be discussed in Section 3.5).

### 3.5. IMU Tightly-Coupled Odometry Calculation

**Figure 6:**
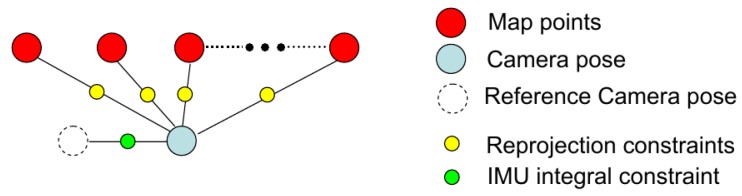
Factor graph for stereo IMU tightly-coupled odometry.

The integration of an IMU motion prior to stereo VO has two advantages: (1) it provides a good initial motion guess for feature guided matching; (2) it gives a motion prior constraint for odometry optimization. We designed a tightly-coupled stereo VO by adding an IMU integral constraint into the 3D-2D re-projection cost non-linear optimization framework. Figure 6 shows the factor graph for the stereo VO; the camera pose tracking w.r.t. the local map can also be seen as a motion-only bundle adjustment. In this graph, map points and reference frame pose are fixed; only the current pose is set free for optimization. The cost function is:

(8) $$\begin{array}{c} \left(R,t\right)=argmin(w(\sum_{i=1}^{N}{∥l_{i}-\pi^{l}\left(P_{i};R,t\right)∥}^{2}\\ +∥r_{i}-\pi^{r}\left(P_{i};R,t\right){∥}^{2})+\left(1-w\right)∥I_{imu}-{\left(R,t\right)}^{T}{∥}^{2}) \end{array}$$

where the current camera pose (R,t) is calculated by minimizing a non-linear re-projection error cost function. The 3D point in the local map is $P_{i}=\left(x_{i},y_{i},z_{i}\right)$; its matched 2D features in the current stereo rig are $l_{i}=\left(u_{i}^{l},v_{i}^{l}\right)$ and $r_{i}=\left(u_{i}^{r},v_{i}^{r}\right)$ for left and right images; $\pi^{l}$ and $\pi^{r}$ are the 3D-2D re-projection model for left and right cameras, respectively. *N* indicates the number of matched features. $I_{imu}$ denotes the IMU motion integral between the current stereo frame and the reference stereo frame. The term $∥I_{imu}-{\left(R,t\right)}^{T}{∥}^{2}$ represents the IMU integral residual. w⊂[0,1) is the weight for the IMU integral constraint.

The optimal solution for the camera pose tracking is obtained by Levenberg–Marquardt iteration:

(9) $$\left(J_{x}^{T}J_{x}+\lambda I\right)\Delta X=-J_{x}\epsilon_{x}$$

where $J_{x}$ and $\epsilon_{x}$ are the Jacobian and residual at current pose *x* for the stereo pose tracking system. It has the form:

(10) $$J_{x}=\left[\begin{array}{c} w(J_{reproj})\\ \left(1-w\right)\left(I_{6\times 6}\right) \end{array}\right]$$

(11) $$\epsilon_{x}=\left[\begin{array}{c} w(\epsilon_{reproj})\\ \left(1-w\right)\left(\epsilon_{imu}\right) \end{array}\right]$$

where $I_{6\times 6}$ is a 6×6 unit matrix. $J_{reproj}$ is the Jacobian for feature re-projection error. $\epsilon_{reproj}$ is feature re-projection error. $\epsilon_{imu}$ indicates the IMU integral residual.

For each map point $P_{i}={\left(Xi,Yi,Zi\right)}^{T}$, its 3D-2D reprojection error $\epsilon_{reproj}\left(i\right)$ is calculated as:

(12) $$\begin{array}{c} \epsilon_{reproj}\left(i\right)=m_{i}-\pi \left(P_{i};R,t\right)\\ =m_{i}-\frac{1}{Z_{c}}\left[\begin{array}{ccc} f_{x} & 0 & u_{0}\\ 0 & f_{y} & v_{0} \end{array}\right]\left[R\left(\begin{array}{c} X_{i}\\ Y_{i}\\ Z_{i} \end{array}\right)+t-\left(\begin{array}{c} B\\ 0\\ 0 \end{array}\right)\right] \end{array}$$

where $m_{i}\in l_{i},r_{i}$ indicates the measured feature coordinate for the left or the right images. $Z_{c}$ is the feature depth by transforming the map point to the left camera coordinate frame. B=0 for the left camera, and B=−baseline for right camera. $f_{x},f_{y},u_{0},v_{0}$ are the stereo intrinsic parameters.

For the optimization, we utilize the minimal parametrization for the camera pose R,t in Lie manifold SE(3) denoted as: $X={\left(\theta_{x},\theta_{y},\theta_{z},t_{x},t_{y},t_{z}\right)}^{T}$. The Jacobian $J_{reproj}\left(i\right)$ for the 3D-2D re-projection error $\epsilon_{reproj}\left(i\right)$ w.r.t. the camera pose *X* is:

(13) $$\begin{array}{c} J_{reproj}\left(i\right)=\left[\begin{array}{c} \frac{\partial \epsilon_{reproj}\left(i\right)}{\partial u_{i}^{l}}\frac{\partial u_{i}^{l}}{\partial X}\\ \frac{\partial \epsilon_{reproj}\left(i\right)}{\partial v_{i}^{l}}\frac{\partial v_{i}^{l}}{\partial X}\\ \frac{\partial \epsilon_{reproj}\left(i\right)}{\partial u_{i}^{r}}\frac{\partial u_{i}^{r}}{\partial X} \end{array}\right]\\ =\left[\begin{array}{cccccc} f_{x}\frac{X_{c}Y_{c}}{Z_{c}^{2}} & -f_{x}\frac{X_{c}^{2}+Z_{c}^{2}}{Z_{c}^{2}} & -f_{x}\frac{Y_{c}}{Z_{c}} & -f_{x}\frac{1}{Z_{c}} & 0 & f_{x}\frac{X_{c}}{Z_{c}^{2}}\\ f_{y}\frac{Y_{c}^{2}+Z_{c}^{2}}{Z_{c}^{2}} & -f_{y}\frac{X_{c}Y_{c}}{Z_{c}^{2}} & -f_{y}\frac{X_{c}}{Z_{c}} & 0 & -f_{y}\frac{1}{Z_{c}} & f_{y}\frac{Y_{c}}{Z_{c}^{2}}\\ f_{x}\frac{X_{c}Y_{c}}{Z_{c}^{2}}-B\frac{Y_{c}}{Z_{c}^{2}} & -f_{x}\frac{X_{c}^{2}+Z_{c}^{2}}{Z_{c}^{2}}+B\frac{X_{c}}{Z_{c}^{2}} & -f_{x}\frac{Y_{c}}{Z_{c}} & -f_{x}\frac{1}{Z_{c}} & 0 & f_{x}\frac{X_{c}}{Z_{c}^{2}}-B\frac{1}{Z_{c}^{2}} \end{array}\right] \end{array}$$

where $P_{c}={\left(X_{c},Y_{c},Z_{c}\right)}^{T}$ is the map point 3D coordinate in the left camera frame, i.e., $P_{c}=RP_{i}+t$. The first two rows are the residual Jacobian w.r.t. the left image, and the last row is for right image.

As a result, for *N* stereo features, the final system Jacobian $J_{x}$ has 3N+6 rows. Additionally, based on the incremental solution ΔX=(Δθ,Δt) from Equation (9), the update of the current camera pose is expressed as:

(14) $$\begin{array}{c} R=\exp\left({\left[\Delta \theta \right]}_{\times }\right)R\\ t=\exp\left({\left[\Delta \theta \right]}_{\times }\right)t+\Delta t \end{array}$$

where ${\left[\Delta \theta \right]}_{\times }$ is the skew-symmetric matrix of the incremental rotation vector Δθ and $\exp\left({\left[\Delta \theta \right]}_{\times }\right)$ is an exponential map.

## 4. Robust Multi-Sensor Fusion Based on a Stochastic Cloning EKF

**Figure 7:**
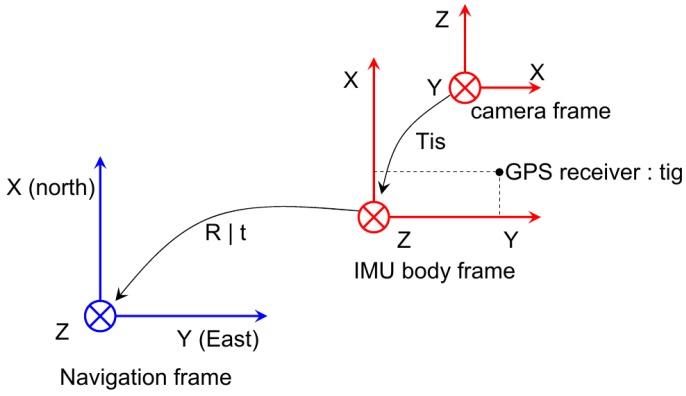
Definition of coordinate frames for EKF state estimation. The navigation frame (or world frame) is a local NED (North-East-Down) frame. The transforms *R* and *t* from the IMU body frame to the navigation frame will be estimated by the EKF. The parameters $T_{is}$ (from the camera frame to the IMU body frame) and $t_{ig}$ (GPS receiver coordinate in IMU body frame) are obtained by calibration.

This section presents an EKF state estimator for the multi-sensor loosely-coupled state estimation. In the EKF, IMU measurements are utilized to propagate the system state and covariance. For the update of the EKF state, both absolute measurements (GPS and barometer) and relative state measurements (stereo VO) are fused. The coordinate systems for the EKF estimator are shown in Figure 7. The navigation frame is a local NED (North-East-Down) frame, and the initial position is determined by the first GPS measurement. The EKF estimates the IMU body frame pose w.r.t. the navigation frame. The transformation from the camera frame to the IMU body frame is denoted as $T_{is}$, and the GPS receiver coordinate in the IMU body frame is $t_{ig}$.

### 4.1. IMU Integration

The IMU sensor measures the tri-axis accelerations and tri-axis angular rates w.r.t. the IMU body frame. The measurements given by the IMU are corrupted by Gaussian noise and a slowly varying bias terms, which must be removed before state estimation processing. Furthermore, the IMU accelerometers measure the force, which must be compensated by gravity. The following continuous-time model expresses the relationship between the IMU measured signals and true ones:

(15) $$\begin{array}{c} \omega_{m}=\omega +b_{g}+n_{g}\\ a_{m}=a+R^{T}g+b_{a}+n_{a} \end{array}$$

where $\omega_{m}\in R^{3}$ and $a_{m}\in R^{3}$ are the measured acceleration and angular rate, respectively. $\omega \in R^{3}$ and $a\in R^{3}$ indicate the true signals. $n_{g}$ and $n_{a}$ are zero-mean Gaussian $N(0,\sigma_{g}^{2})$ and $N(0,\sigma_{a}^{2})$; $b_{g}\in R^{3}$ and $b_{a}\in R^{3}$ are slowly varying bias terms for the accelerometer and gyroscope, respectively. Additionally, $g\in R^{3}$ is gravity acceleration; the rotation matrix R∈SO(3) indicates the current IMU pose w.r.t. the navigation frame.

The estimated angular rate and acceleration rate are denoted as $\hat{\omega }\in R^{3}$, $\hat{a}\in R^{3}$, respectively. Additionally, the estimated bias terms for angular rate and acceleration are ${\hat{b}}_{g}$ and ${\hat{b}}_{a}$; we have:

(16) $$\begin{array}{c} \hat{\omega }=\omega_{m}-{\hat{b}}_{g},\hat{a}=a_{m}-{\hat{b}}_{a} \end{array}$$

Denote $\delta b_{g}=b_{g}-{\hat{b}}_{g}$, $\delta b_{a}=b_{a}-{\hat{b}}_{a}$ as the bias errors between the true bias $b_{g}$, $b_{a}$ and the estimated bias ${\hat{b}}_{g}$, ${\hat{b}}_{a}$, and the slowly varying motion for bias errors are modeled as:

(17) $$\begin{array}{c} \dot{\delta b_{g}}=r_{g},\dot{\delta b_{a}}=r_{a} \end{array}$$

where $r_{g}∼N\left(0,\sigma_{rg}^{2}\right)$ and $r_{a}∼N\left(0,\sigma_{ra}^{2}\right)$ are zero-mean Gaussian.

Based on the above IMU kinematic model, the discrete IMU integral equations are:

(18) $$\begin{array}{c} p\left(k+1\right)=p\left(k\right)+v\left(k\right)dt+\frac{1}{2}\hat{a}{dt}^{2}\\ v_{b}\left(k+1\right)=v_{b}\left(k\right)+\left(\hat{a}-{\left[\hat{\omega }\right]}_{\times }v_{b}\left(k\right)\right)dt\\ R\left(k+1\right)=R\left(k\right)\exp\left({\left[\hat{\omega }dt\right]}_{\times }\right) \end{array}$$

where $p\left(k\right)\in R^{3}$ indicates the three D.O.F position w.r.t. the navigation frame at instant *k*. $v_{b}\left(k\right)$ is the velocity defined in the IMU body frame, and R(k)∈SO(3) is the rotation matrix w.r.t. the navigation frame. ${\left[\hat{\omega }dt\right]}_{\times }$ is a skew-symmetric matrix of the angular rate integral rotation vector $\hat{\omega }dt$; $\exp\left({\left[\hat{\omega }dt\right]}_{\times }\right)$ is an exponential map in the Lie manifold SO(3). dt is the IMU sampling time.

### 4.2. EKF State Definition and Jacobians

Based on the IMU integral equations and bias error model, the EKF system state *S* is defined as:

(19) $$\begin{array}{c} S={\left(p,\delta \theta ,v_{b},\delta b_{g},\delta b_{a}\right)}^{T}\in R^{15} \end{array}$$

where $p\in R^{3}$ indicates position w.r.t. the navigation frame, $\delta \theta \in R^{3}$ is the error rotation vector w.r.t. the IMU body frame, $v_{b}\in R^{3}$ is the velocity w.r.t. the IMU body frame and $\delta b_{g}\in R^{3}$, $\delta b_{a}\in R^{3}$ are the current bias error terms.

The estimated rotation matrix is defined as $\hat{R}\in \mathrm{SO}\left(3\right)$, so the true rotation matrix R∈SO(3) after the rotation error compensation is calculated by matrix right multiplication:

(20) $$R=\hat{R}\exp\left({\left[\delta \theta \right]}_{\times }\right)$$

where ${\left[\delta \theta \right]}_{\times }$ is skew-symmetric matrix of error rotation vector δθ.

Based on the above system state definition, the system state dynamics $\dot{S}$ is derived as:

(21) $$\begin{array}{c} \dot{p}=\hat{R}\exp\left({\left[\delta \theta \right]}_{\times }\right)v_{b}\\ \dot{\delta \theta }=\exp\left({\left[\delta \theta \right]}_{\times }\right)\left(\hat{\omega }-\delta b_{g}-n_{g}\right)\\ \dot{v_{b}}=-{\left[\hat{\omega }-\delta b_{g}-n_{g}\right]}_{\times }v_{b}+{\left(\hat{R}\exp\left({\left[\delta \theta \right]}_{\times }\right)\right)}^{T}g+\hat{a}-\delta b_{a}-n_{a}\\ \dot{\delta b_{g}}=r_{g}\\ \dot{\delta b_{a}}=r_{a} \end{array}$$

Therefore, the Jacobian matrix $\frac{d\dot{S}}{dS}\in R^{15\times 15}$ for the system dynamics is obtained as:

(22) $$\frac{\partial \dot{S}}{\partial S}=\left(\begin{array}{ccccc} 0_{3\times 3} & -\hat{R}{\left[v_{b}\right]}_{\times } & \hat{R}\exp\left({\left[\delta \theta \right]}_{\times }\right) & 0_{3\times 3} & 0_{3\times 3}\\ 0_{3\times 3} & -{\left[\hat{\omega }-\delta b_{g}-n_{g}\right]}_{\times } & 0_{3\times 3} & -\exp\left({\left[\delta \theta \right]}_{\times }\right) & 0_{3\times 3}\\ 0_{3\times 3} & {\left[{\hat{R}}^{T}g\right]}_{\times } & -{\left[\hat{\omega }-\delta b_{g}-n_{g}\right]}_{\times } & -{\left[v_{b}\right]}_{\times } & -I_{3\times 3}\\ 0_{3\times 3} & 0_{3\times 3} & 0_{3\times 3} & 0_{3\times 3} & 0_{3\times 3}\\ 0_{3\times 3} & 0_{3\times 3} & 0_{3\times 3} & 0_{3\times 3} & 0_{3\times 3} \end{array}\right)$$

where $I_{3\times 3}$ denotes the 3×3 identity matrix and $0_{3\times 3}$ denotes the 3×3 zero matrix.

The system state noise input consists of IMU measurement noise and bias error noise, that is:

(23) $$W={\left(n_{g},n_{a},r_{g},r_{a}\right)}^{T}\in R^{12}$$

As a result, the Jacobian matrix $\frac{d\dot{S}}{dW}\in R^{15\times 12}$ w.r.t. the system noise is:

(24) $$\frac{\partial \dot{S}}{\partial W}=\left(\begin{array}{cccc} 0_{3\times 3} & 0_{3\times 3} & 0_{3\times 3} & 0_{3\times 3}\\ -I_{3\times 3} & 0_{3\times 3} & 0_{3\times 3} & 0_{3\times 3}\\ -{\left[v_{b}\right]}_{\times } & -I_{3\times 3} & 0_{3\times 3} & 0_{3\times 3}\\ 0_{3\times 3} & 0_{3\times 3} & I_{3\times 3} & 0_{3\times 3}\\ 0_{3\times 3} & 0_{3\times 3} & 0_{3\times 3} & I_{3\times 3} \end{array}\right)$$

Based on the relationship between the continuous-time and discrete-time systems, the final Jacobians for state covariance propagation are:

(25) $$\begin{array}{c} J_{S}=\frac{\partial \dot{S}}{\partial S}dt+I_{15\times 15},J_{W}=\frac{\partial \dot{S}}{\partial W}dt \end{array}$$

**Remark** **1.** In this section, the rotational error δθ is defined in a local coordinate system (current IMU body frame). As a result, the covariance $\Sigma_{\delta \theta }^{2}$ is also w.r.t. the current local frame. On the basis of the adjoint map of SO(3), the error rotate vector δθ can be expressed in global navigation frame $\hat{R}\exp\left({\left[\delta \theta \right]}_{\times }\right)=\exp\left({\left[\hat{R}\delta \theta \right]}_{\times }\right)\hat{R}$. The covariance for error rotation w.r.t. the navigation frame is $\hat{R}\Sigma_{\delta \theta }^{2}{\hat{R}}^{T}$.

### 4.3. Treatment of VO Relative State Measurement Using Delayed State Stochastic Cloning

Our state estimation system utilizes both absolute state measurements (GPS provides absolute position and velocity measurement in the NED coordinate system; the barometer provides absolute state measurement for altitude) and the relative six D.O.F pose measurement (between the two stereo frames) provided by long-range stereo VO. To deal-with both absolute and relative state measurements, the system state defined in Equation (19) is further augmented by stochastic cloning of a delayed pose $p_{l},\delta \theta_{l}$, which is updated with the previous VO measurement, namely:

(26) $$\begin{array}{c} \tilde{S}={\left(S^{T},p_{l},\delta \theta_{l}\right)}^{T}\in R^{21} \end{array}$$

During the system state propagation, the delayed pose $p_{l},\delta \theta_{l}$ is kept as constant; that means $\dot{p_{l}}=0$ and $\dot{\delta \theta_{l}}=0$. Therefore, the Jacobians for the augmented state $\tilde{S}$ are:

(27) $$\tilde{J_{S}}=\left(\begin{array}{cc} J_{S} & 0_{15\times 6}\\ 0_{6\times 15} & I_{6\times 6} \end{array}\right)\in R^{21\times 21}$$

(28) $$\tilde{J_{W}}=\left(\begin{array}{c} J_{W}\\ 0_{6\times 12} \end{array}\right)\in R^{21\times 12}$$

The augmented state covariance is denoted as $\tilde{P}\left(k\right)\in R^{21\times 21}$. Accordingly, the covariance propagation for the state augmented system is given as:

(29) $$\begin{array}{c} \tilde{P}\left(k+1|k\right)=\tilde{J_{S}}\tilde{P}\left(k\right){\tilde{J_{S}}}^{T}+\tilde{J_{W}}Q\left(K\right){\tilde{J_{W}}}^{T} \end{array}$$

For the system initialization, the initial system state covariance is of the form:

(30) $$\tilde{P}\left(0\right)=\left(\begin{array}{ccccccc} \Sigma_{p}^{2} & 0 & 0 & 0 & 0 & \Sigma_{p}^{2} & 0\\ 0 & \Sigma_{\theta }^{2} & 0 & 0 & 0 & 0 & \Sigma_{\theta }^{2}\\ 0 & 0 & \Sigma_{vb}^{2} & 0 & 0 & 0 & 0\\ 0 & 0 & 0 & \Sigma_{bg}^{2} & 0 & 0 & 0\\ 0 & 0 & 0 & 0 & \Sigma_{ba}^{2} & 0 & 0\\ \Sigma_{p}^{2} & 0 & 0 & 0 & 0 & \Sigma_{p}^{2} & 0\\ 0 & \Sigma_{\theta }^{2} & 0 & 0 & 0 & 0 & \Sigma_{\theta }^{2} \end{array}\right)$$

Long-range stereo VO generates the relative six D.O.F motion measurement between the two visual frames. The relative measurement model is defined as:

(31) $$\begin{array}{c} \Delta p=\exp\left(-{\left[\delta \theta_{l}\right]}_{\times }\right)R_{l}^{T}\left(p-p_{l}\right)\\ \Delta \theta =\log(\exp\left(-{\left[\delta \theta_{l}\right]}_{\times }\right)R_{l}^{T}\hat{R}\exp\left({\left[\delta \theta \right]}_{\times }\right)) \end{array}$$

where $\Delta p\in R^{3}$ is a position increment from the current pose $p,\hat{R}$ to the delay pose $p_{l},R_{l}$ and $\Delta \theta \in R^{3}$ is the rotation increment. $R_{l}\in \mathrm{SO}\left(3\right)$ is the rotation matrix for previous visual updated orientation (i.e., the delayed state orientation), and $\delta \theta_{l}$ indicates the error rotation vector for the delayed state. $\hat{R}\in \mathrm{SO}\left(3\right)$ is the rotation matrix for the current orientation, and δθ is the current error rotation vector. The matrix logarithm $\log(R_{l}^{T}\hat{R})$ maps the rotation matrix $R_{l}^{T}\hat{R}$ to a rotation vector.

For Jacobians with a relative translation Δp w.r.t. system state *S*, we have:

(32) $$\begin{array}{c} \frac{\partial \Delta p}{\partial p}=\exp\left(-{\left[\delta \theta_{l}\right]}_{\times }\right){|}_{\delta \theta_{l}=0}R_{l}^{T}=R_{l}^{T}\\ \frac{\partial \Delta p}{\partial p_{l}}=-\exp\left(-{\left[\delta \theta_{l}\right]}_{\times }\right){|}_{\delta \theta_{l}=0}R_{l}^{T}=-R_{l}^{T}\\ \frac{\partial \Delta p}{\partial \delta \theta_{l}}=\frac{\partial \exp\left(-{\left[\delta \theta_{l}\right]}_{\times }\right)}{\partial \delta \theta_{l}}{|}_{\delta \theta_{l}=0}R_{l}^{T}\left(p-p_{l}\right)={\left[R_{l}^{T}\left(p-p_{l}\right)\right]}_{\times } \end{array}$$

where the derivative $\frac{\partial \Delta p}{\partial \delta \theta_{l}}$ is derived based on the first-order Taylor expansion for the exponential map at $\delta \theta_{l}=0$, that is $\exp\left(-{\left[\delta \theta_{l}\right]}_{\times }\right){|}_{\delta \theta_{l}=0}\approx 1-{\left[\delta \theta_{l}\right]}_{\times }$. Additionally, the anti-commutativity rule for skew-symmetric matrix, namely: ${\left[\delta \theta_{l}\right]}_{\times }R_{l}^{T}\left(p-p_{l}\right)=-{\left[R_{l}^{T}\left(p-p_{l}\right)\right]}_{\times }\delta \theta_{l}$.

The Jacobians for the Δθ are computed as:

(33) $$\begin{array}{c} \frac{\partial \Delta \theta }{\partial \delta \theta }=\frac{\partial \log(R_{l}^{T}\hat{R}\exp\left({\left[\delta \theta \right]}_{\times }\right))}{\partial \delta \theta }{|}_{\delta \theta =0}=Adj\left(R_{l}^{T}\hat{R}\right)=R_{l}^{T}\hat{R}\\ \frac{\partial \Delta \theta }{\partial \delta \theta_{l}}=\frac{\partial \log(\exp\left(-{\left[\delta \theta_{l}\right]}_{\times }\right)R_{l}^{T}\hat{R})}{\partial \delta \theta_{l}}{|}_{\delta \theta_{l}=0}=-Adj\left(I_{3\times 3}\right)=-I_{3\times 3} \end{array}$$

where Adj(R) is the adjoint map in R∈SO(3), and it has the property of Adj(R)=R. The derivative for the matrix logarithm is derived by the first-order approximation of Campbell–Baker–Hausdorff formula. For the logarithm map derivative with a unified form like $\frac{\partial \log(A\exp\left({\left[\delta \theta \right]}_{\times }\right))B}{\partial \delta \theta }{|}_{\delta \theta =0}$, its derivative can be estimated by Adj(A) under the condition that AB approximates the identity. More details for the logarithm derivation can be found in reference [26].

As a result, the VO relative measurement Jacobian is expressed as:

(34) $$H_{vo}=\left(\begin{array}{cccc} R_{l}^{T} & 0_{3\times 12} & -R_{l}^{T} & {\left[R_{l}^{T}\left(p-p_{l}\right)\right]}_{\times }\\ 0_{3\times 3} & R_{l}^{T}\hat{R} & 0_{3\times 12} & -I_{3\times 6} \end{array}\right)$$

Denote the VO relative measurement as ${\left(\Delta p_{vo},\Delta \theta_{vo}\right)}^{T}$; the measurement residual is given by:

(35) $$\tilde{r}=\left(\begin{array}{c} \Delta p_{vo}-\Delta p\\ \Delta \theta_{vo}⊖\Delta \theta \end{array}\right)$$

where the rotational vector residual $\Delta \theta_{vo}⊖\Delta \theta$ is defined as: $log(\Delta R^{-1}\Delta R_{vo})$. $\Delta R=\exp\left({\left[\Delta \theta \right]}_{\times }\right)$ is the predicted rotation matrix from the current state to the delayed state. Additionally, the $\Delta R_{vo}=\exp\left({\left[\Delta \theta_{vo}\right]}_{\times }\right)$ is the VO measured one.

It is worthwhile to note that, after each VO relative measurement update, the delayed portion vector of the state $p_{l},\delta \theta_{l}$ is set equal to the current updated pose p(k+1),δθ(k+1), and the state covariance matrix is updated by “cloning” the corresponding covariance blocks from the current state covariance to delayed pose covariance. To update the EKF state, we should first transform the VO measurement from the visual frame to the IMU body frame using the visual-IMU relative pose calibration $T_{is}$; suppose the VO measurement in visual frame is $Z_{s}$; its corresponding measurement in the IMU body frame is:

(36) $$\begin{array}{c} Z_{i}=T_{is}Z_{s}{T_{is}}^{-1} \end{array}$$

The update of the EKF state is standard, that is:

(37) $$\begin{array}{c} K=\tilde{P}\left(k+1|k\right)H^{T}{\left(\tilde{P}\left(k+1|k\right)H^{T}+R\right)}^{-1}\\ \tilde{S}\left(k+1\right)=\tilde{S}\left(k\right)+K\tilde{r} \end{array}$$

The EKF covariance update uses Joseph’s form to avoid the negative definition, that is:

(38) $$\begin{array}{c} \tilde{P}\left(k+1\right)=\left(I-KH\right)\tilde{P}\left(k+1|k\right){\left(I-KH\right)}^{T}+KRK^{T} \end{array}$$

**Remark** **2.** After the VO relative measurement update, the updated covariance $\tilde{P}\left(k+1\right)$ should keep two important properties: (1) it should be lower than IMU state propagation covariance $\tilde{P}\left(k+1|k\right)$ since VO information is available to the system; and (2) it must be increased compared with previous error covariance $\tilde{P}\left(k\right)$. Otherwise, the absolute measurement (GPS and barometer) will lose the ability of updating the state estimation. Compared with pseudo absolute measurement VO update approaches, the covariance using delayed state cloning EKF can meet the two properties. This will be verified in Section 5.3 and Section 5.4.

### 4.4. Update of EKF State Using Absolute State Measurements

GPS provides absolute position and velocity measurement in the NED frame system; suppose the heading of the initial EKF navigation frame is aligned with the NED frame; the GPS measurement model is:

(39) $$Z_{gps}=\left[\begin{array}{c} p+\hat{R}\exp\left({\left[\delta \theta \right]}_{\times }\right)t_{ig}\\ \hat{R}\exp\left({\left[\delta \theta \right]}_{\times }\right)\left(v_{b}+{\left[\hat{\omega }-\delta b_{g}\right]}_{\times }t_{ig}\right) \end{array}\right]$$

where $t_{ig}\in R^{3}$ is the translation from the GPS receiver to the IMU body frame, as explained in Figure 7. The GPS measurement Jacobian is derived as:

(40) $$H_{gps}=\left(\begin{array}{ccccc} I_{3\times 3} & -\hat{R}{\left[t_{ig}\right]}_{\times } & 0_{3\times 3} & 0_{3\times 3} & 0_{3\times 3}\\ 0_{3\times 3} & -\hat{R}{\left[v_{b}\right]}_{\times } & \hat{R} & {\left[t_{ig}\right]}_{\times } & 0_{3\times 3} \end{array}\right)$$

Since GPS measurement in altitude has a large uncertainty, the GPS height and velocity in altitude are not utilized to update the EKF state. Only the position and velocity for north and east are kept as GPS measurements, namely $Z_{gps}={\left(p_{n},p_{e},v_{n},v_{e}\right)}^{T}\in R^{4}$. Consequently, the third and the sixth rows for the GPS Jacobian $H_{gps}$ are also removed.

We utilized the “GPS health status”, which reports how many satellites can be seen by the receiver, to determine the current GPS measurement covariance. For bad “GPS health status”, GPS will report a large covariance. It is worth mentioning that the $\chi^{2}$ test at 0.95 is utilized to verify the compatibility between current GPS measurement and the system predicted state. If GPS measurement “jumps” due to perturbation (e.g., multipath), the system will reject the GPS measurement automatically. In fact, all of the sensor measurements are firstly checked by the $\chi^{2}$ test before they are utilized for state estimation. As a result, the EKF state estimator is robust to any sensor failures.

The barometer provides absolute altitude measurements w.r.t. the navigation frame. The navigation frame is a local NED frame, so the barometer measures the negative altitude w.r.t. the NED coordinate. As a result, the barometer measurement model is:

(41) $$Z_{baro}=-p_{d}$$

where $p_{d}$ denotes the *z* component for current position. Its Jacobian is:

(42) $$H_{baro}=\left(\begin{array}{cccc} 0 & 0 & -1 & 0_{1\times 18} \end{array}\right)$$

**Figure 8:**
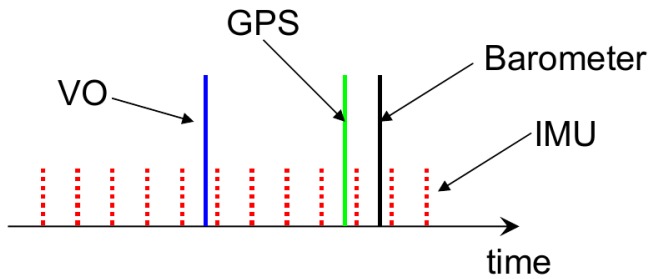
A ring buffer is utilized to keep 2 s of incoming sensor information. Due to the image transmission and Visual Odometry (VO) calculation delay, the newly-arriving VO measurements do not have the newest time stamp. The VO will update the EKF state on the VO time, and the subsequent IMU integral is re-integrated to predict the current state.

For the EKF implementation, a ring buffer with a 2-s time is kept to save all of the incoming sensor data. As shown in Figure 8, when a new VO measurement arrives, its time stamp is usually not the most up to date due to the image transmission and the stereo VO calculation delay. For this case, after the update of the EKF state on the VO time stamp, all of the subsequent IMU integral should be re-integrated to re-predict the current state. The same processing is also carried out for GPS and barometric measurements. To further decrease the computational cost of IMU re-integration, the IMU pre-integral technique in the IMU body frame can be utilized.

**Remark** **3.** After an absolute measurement update of the EKF state by the GPS or barometer, both the current pose covariance $\Sigma_{p}^{2},\Sigma_{\theta }^{2}$ and the delayed pose covariance $\Sigma_{p_{l}}^{2},\Sigma_{\theta_{l}}^{2}$ are decreased. Furthermore the current pose covariance $\Sigma_{p}^{2},\Sigma_{\theta }^{2}$ should be higher than the delayed pose covariance $\Sigma_{p_{l}}^{2},\Sigma_{\theta_{l}}^{2}$. Otherwise, the VO relative measurement will lose the ability to update of the EKF state.

## 5. Results

### 5.1. Experimental System

**Figure 9:**
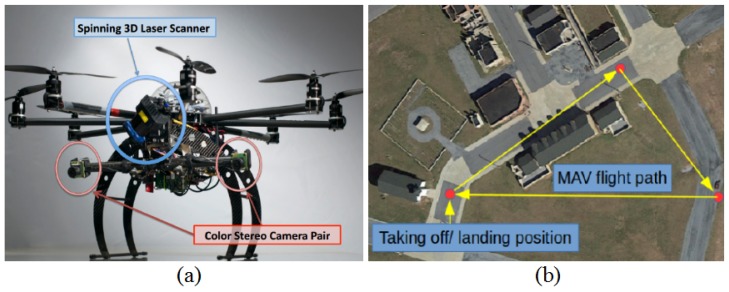
Our MAV for the dataset recording and experimental environment. (**a**) MAV developed by our team for the state estimation experiments; (**b**) experimental scenario from Google Earth and the schematic flight trajectory for the MAV.

**Figure 10:**
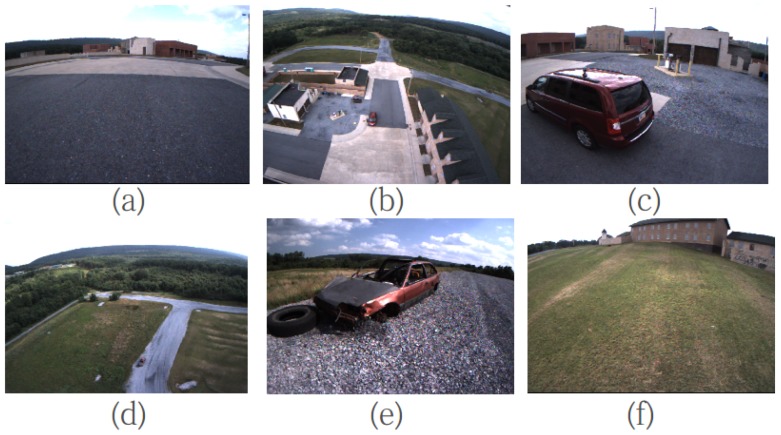
Some typical images from the experiment: (**a**) the MAV taking off; (**b**,**d**) the high-altitude flight; (**c**,**e**) low-altitude flight to capture the images for the two cars’ stereo reconstruction; and (**f**) the similar texture environment.

In this section, we present the experimental results for the proposed technique. Figure 9a shows the MAV developed by our team for the state estimation experiments; some additional sensors, including a commercial GPS, stereo camera, an IMU (Microstrain 3DM-GX3-35 [27]) a barometer and a spinning laser scanner, were carried onboard by the MAV for dataset gathering. All the sensors are hardware triggered, so the timestamps for different onboard sensors have the same time reference. The IMU was recorded at 100 Hz; the barometer rate was 7 Hz; GPS was 4 Hz; and stereo was 10 Hz. For the forward facing stereo (about $-15^{\circ }$ pitch) with a 0.41 m static baseline, its effective short-range measurement is 13.41 m with a 10-pixel stereo disparity. The datasets are gathered in Fort Indiantown Gap, Pennsylvania. Figure 9b shows the experimental scenario from Google Earth and the schematic flight trajectory for the MAV. For the dataset, the MAV flies 12 min with aggressive six D.O.F motions. Some topical scenarios for this experiments are shown in Figure 10.

### 5.2. Performance of IMU Tightly-Coupled Long-Range Stereo Odometry

**Figure 11:**
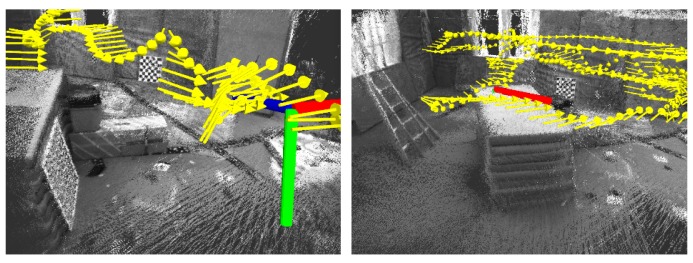
3D environments reconstructed by our stereo VO for the EuRoC (European Robotics Challenge) “room-1” dataset.

We first utilized the EuRoC (European Robotics Challenge) dataset [28] to evaluate the proposed long-range stereo VO. Then, we compared the performance of long-range VO with that of the state-of-the-art sparse feature ORBSLAM 2.0. For fair comparison, the loop-closing detection thread and global bundle adjustment for ORBSLAM2.0 are deactivated. The EuRoc dataset contains nine stereo-IMU datasets recorded by a quadrocopter in three different indoor environments. We utilize the six datasets in VICON [29] room due to the six D.O.F ground truth being provided for the evaluation. The proposed long-range VO and long-range VO tightly coupled with IMU and ORBSLAM 2.0 were tested and compared. In the experiments, we recorded the odometry outputs, including timestamp, position and orientation quaternion. Furthermore, the VICON data are used as the corresponding ground truth. Relative Pose Error (RPE) is used as the evaluation measure. For the IMU integral, the first one second of the IMU dataset is filtered and used for calculating the initial roll, pitch and gravity acceleration with respect to the inertial frame. Figure 11 shows the 3D environments reconstructed by our stereo VO for the “room-1” dataset.

**Figure 12:**
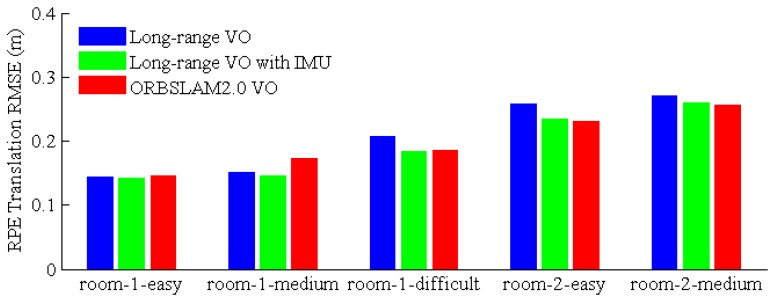
Relative Pose Error (RPE) evaluation results.

Figure 12 gives the RPE evaluation results. From the results, the proposed VO shows similar performance as the ORBSLAM 2.0. For the last dataset (room 2-difficult), all three approaches failed to track the pose at 67 s due to the serious image motion blur and low texture (so, we do not report the result for the last dataset). The VICON room datasets were recorded in relatively small indoor environments; the proposed VO works with pure stereo mode for most situations. Furthermore, the vehicle flies around the room several times for each dataset, so ORBSLAM 2.0 utilizes the built map to localize the vehicle, namely with the localization mode. In comparison, our VO is a sliding-window VO for the onboard memory and computing resource consideration; only some key-frames and their corresponding map points are kept for pose tracking and local BA. As a result, our VO works with exploration mode, which is more difficult than localization mode.

**Figure 13:**
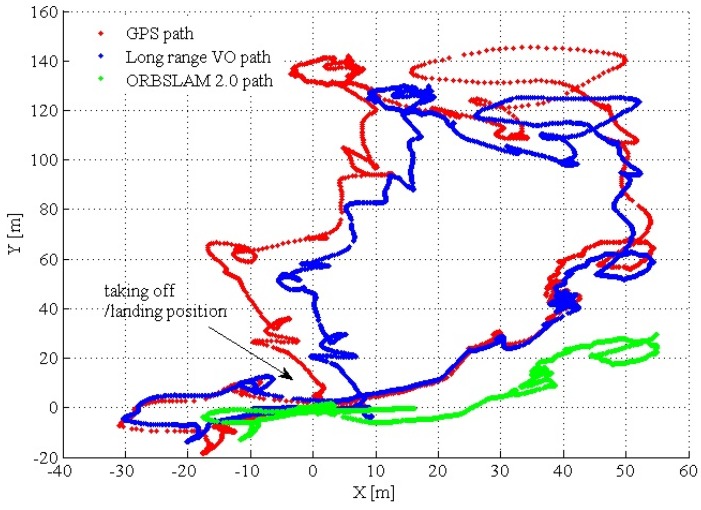
Comparison of the GPS path, proposed long-range VO path and ORBSLAM 2.0 path. The MAV takes off at position (0,0). At this position, we recorded the initial GPS and barometric measurements as the offsets for the subsequence ground truth measurements. The initial MAV heading is aligned with the NED coordinate. ORBSLAM 2.0 (also with IMU tightly coupled) fails at 400 s of the dataset.

**Figure 14:**
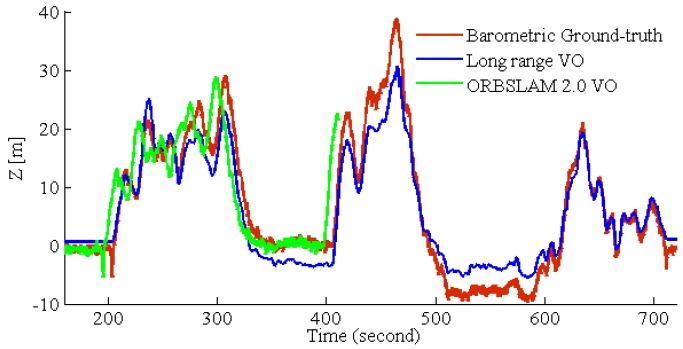
Comparison of long-range VO path and ORBSLAM 2.0 path in the altitude direction. ORBSLAM 2.0 fails at 400 seconds of the dataset. At this time, the MAV altitude increases sharply, so ORBSLAM 2.0 cannot deal with this long-range high-altitude case.

To test the long-range performance, the dataset recorded by our MAV is utilized. For this experiment, GPS and the barometer were utilized as the ground truth. The results are shown in Figure 13. For ORBSLAM2.0 VO, it easily fails to track aggressive MAV motion, so we also integrate the same IMU tightly-coupled strategy (Section 3.5) in ORBSLAM 2.0 VO. The altitude estimation results are shown in Figure 14; ORBSLAM 2.0 VO fails at 400 s of the dataset. At this time, the MAV altitude is sharply increasing, so ORBSLAM 2.0 VO cannot deal with this long-range high-altitude case. The path RMSE is listed in Table 1. The first row is long-range VO RMSE before the ORBSLAM 2.0 VO fails; the last row is the path RMSE for the entire MAV 12-min flights.

### 5.3. Performance of Multi-Sensor Fusion State Estimation

**Figure 15:**
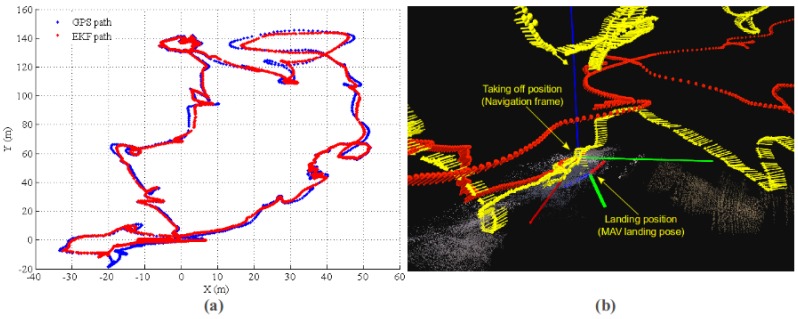
EKF state estimation result. (**a**) EKF estimation path in the X-Y plane; the MAV takes off at (0,0) and comes back after 12 min of flight; (**b**) local details for EKF state estimation. The yellow arrow sequence is the 3D path from EKF, and the red sequence is the GPS path. Clearly, GPS reports bad positioning information in the altitude direction.

With the same dataset, we tested the multi-sensor fusion state estimation performance. The result of MAV path estimation is shown in Figure 15. In this experiment, we utilized the VO pseudo absolute measurement update of EKF state [4,19] for the first 220 s. Then, the VO measurement is switched to relative mode, as discussed in Section 4.3. The purpose of using pseudo absolute measurement for the first 220 s is two-fold: (1) the proposed long-range VO can provide absolute pose measurement w.r.t. local map; also, the VO absolute pose drifts slowly over time, as the result shown in Section 5.1; it can be used for short-term absolute update of EKF state for the initial phase; (2) since the VO absolute update is used for the first 220 s, the system estimation will be overconfident. Other absolute measurement sensors with larger uncertainty than that of VO absolute measurement will be prohibited for system state estimation. This experiment will show this effect by comparing the MAV path estimation consistency.

**Figure 16:**
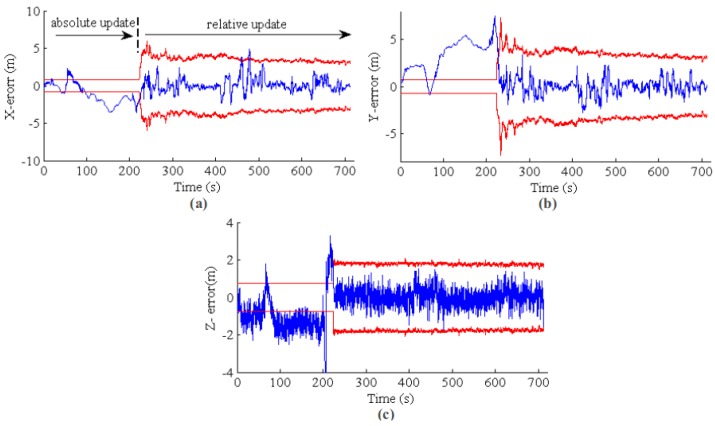
EKF state estimation error and consistency. (**a**–**c**) The results for *X*, *Y* and *Z*, respectively. The blue line is the position error, and the red line is the 3σ error band from the state covariance. Because VO absolute measurement is fused in the initial phase, the state estimation is overconfident and inconsistent. Furthermore, the GPS and barometric measurements are prohibited from updating the EKF state. After the VO is switched to the relative measurement model, the EKF state becomes consistent, and the error is bounded in the 3σ band.

Figure 16 plots the state estimation results for MAV position. The blue line is the EKF positioning error, and the red line is the 3σ error band from EKF state covariance. Because VO absolute measurement is fused in the initial phase, the system covariance from EKF almost kept constant (or a little bit decreasing), and the state estimation is inconsistent compared with the GPS (and barometric) ground truth. In fact, the position error in the X and Ydirections are mainly from the inaccurate initial MAV heading w.r.t. the NED coordinate. However, the initial heading cannot be corrected because GPS is prohibited from updating the EKF state. After the VO is switched to the relative model, the EKF becomes consistent, and the estimation error is bounded in the 3σ error band.

### 5.4. Performance of GPS Outage Situations

**Figure 17:**
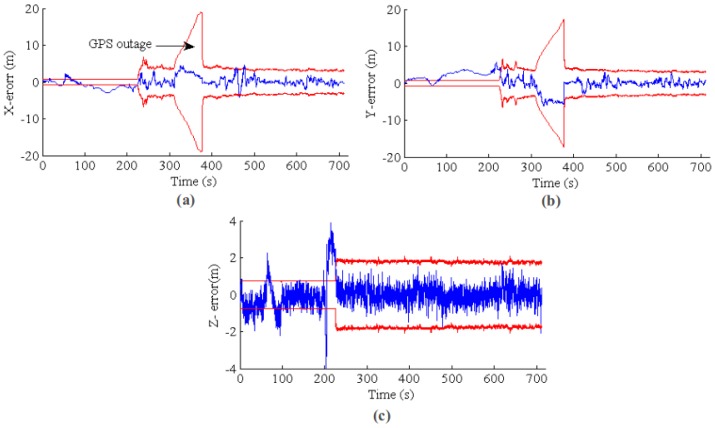
EKF state estimation for GPS outage from 300 s to 360 s. The blue line is the estimation error, and the red line is the 3*σ* error band. As expected, the error covariance is slowly increased by fusing VO relative information. Furthermore, the state estimation is consistent and accurate. (**a**–**c**) The results for *X*, *Y* and *Z*, respectively.

**Figure 18:**
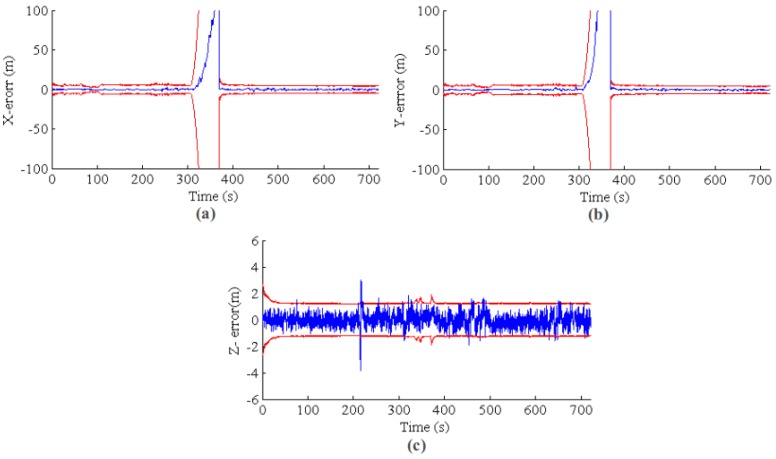
EKF state estimation without the VO update for GPS outage from 300 s to 360 s. Both the error and error covariance are sharply increasing due to the noisy IMU integral. (**a**–**c**) The results for *X*, *Y* and *Z*, respectively. The blue line is the position error, and the red line is the 3σ error band from the state covariance.

A natural question for multi-sensor fusing is “why do we fuse VO for state estimation?”. In this section, the VO information fusion performance for GPS outage situations is investigated. To simulate GPS outage, we do not use GPS for the update of the EKF state in some time periods. Furthermore, the state estimation performance with and without VO fusing are compared. In the first group of experiments, GPS is deactivated for 1 min (GPS lost at 300 s and recovered at 360 s). For the second group of experiments, the GPS outage is relatively long term (about 120 s); we turned off the GPS update at 500 s and never activated it again. The results for the first group of experiments are shown in Figure 17 and Figure 18 for GPS outage with/without VO fusing, respectively. In Figure 17, when the GPS was lost at 300 s, the system fuses the IMU, barometer and VO relative measurement. As expected, the error covariance is slowly increased by fusing VO relative motion information. Furthermore, the state estimation is still consistent and accurate. By comparison, both of error and error covariance are sharply increased without VO fusing. The main reason for the fast drift without VO lies in the noisy IMU measurement. The IMU signal is corrupted by vibration when the MAV motors are powered, especially for the acceleration. Accordingly, the system velocity smooth estimation can only be kept for a short time and will drift quickly by the accumulating IMU acceleration noise. For the altitude direction, both with and without VO can report reasonable results since barometric absolute measurement is provided.

**Figure 19:**
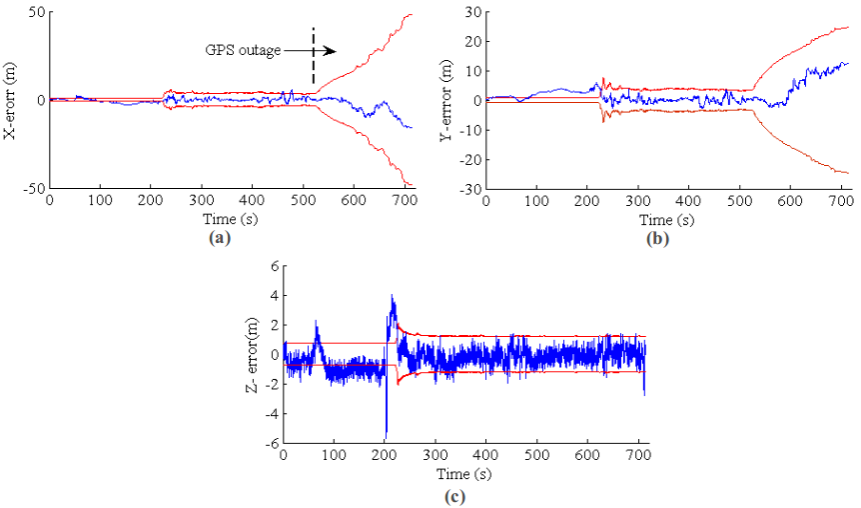
EKF state estimation for a long-term GPS outage from 500 s to the end. The blue line is the estimation error, and the red line is the 3*σ* error band. (**a**–**c**) The results for *X*, *Y* and *Z*, respectively.

**Figure 20:**
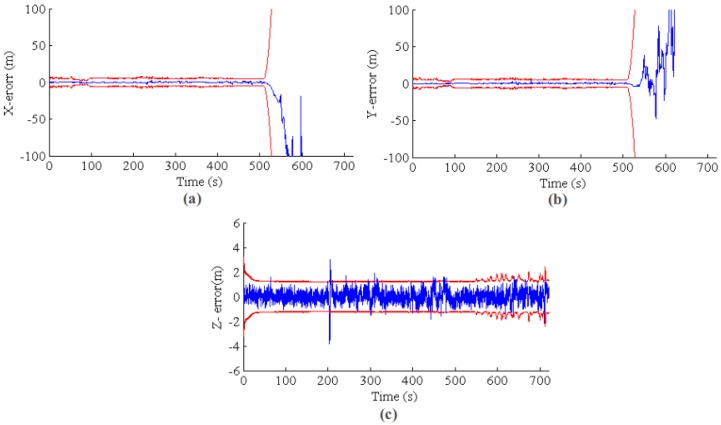
EKF state estimation without VO update for GPS outage from 500 s to the end. Both of the error and error covariance are sharply increased, and state estimation becomes unusable. (**a**–**c**) The results for *X*, *Y* and *Z*, respectively. The blue line is the position error, and the red line is the 3σ error band from the state covariance.

**Figure 21:**
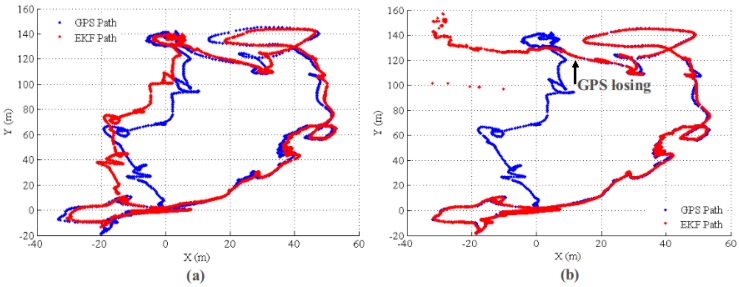
Estimated paths with/without VO update. The GPS outage starts at 500 s. (**a**) Path with VO relative update; (**b**) path without VO relative update.

The results for the second group of experiments are shown in Figure 19 and Figure 20 for GPS outage with and without VO fusion, respectively. The results are similar to that of the first experiments. For the state estimation with VO measurements, the state error and covariance are increasing smoothly; while the state estimation information becomes unusable without a VO relative update. Figure 21 shows the estimated paths for with and without VO measurements update, respectively. It is clear that the EKF state estimator with VO update can generate a smooth, low drift result after the GPS is lost. For quantitative comparison, the RMSEs for the two group experiments are listed in Table 2.

### 5.5. Performance of Timing

All of the experiments were performed offline using the recorded dataset for detailed performance analysis. We have tested the “timing performance” for the state estimation system both on a desktop PC (Intel i7, 2.8 GHZ) and small-sized onboard ARM-based computers (NVIDIA Jetson TX1 [30] and low-cost Odroid XU4 [31]).

The EKF can run in real time both for a desktop PC and two kinds of onboard ARM-based computers. The EKF algorithm is currently developed for ROS (Robot Operation System). It only requires 2 to 3 ms (including state propagation, update, rolling-buffer recalculation and state publication) even for the slowest low-cost Odroid XU4. The long-range stereo VO can run 20 Hz on the desktop PC (so, the proposed VO can run in real time if the Intel i7 onboard computer is utilized for MAV), 7 Hz on the NVIDIA Jetson TX1 onboard computer and 5 Hz on the slowest low-cost Odroid XU4. The most time-consuming part for stereo VO is feature detection, descriptor extraction and stereo matching. For the Intel i7 computer, it requires about 28 ms. In comparison, for the slowest Odroid XU4, it requires about 144 ms (five-times slower), because the current stereo VO implementation uses some functions from OpenCV, which is much slower than that running on the Intel i7 computer. Next, we will focus on code optimization to reduce stereo VO time consumption on the low-cost Odroid XU4.

## 6. Conclusions

In this paper, we present an EKF multi-sensor state estimator by fusing long-range VO, GPS, IMU and a barometer for MAV navigation in both GPS-available and GPS-denied environments.

Firstly, we derived a new long-range stereo VO. The main reason for using the stereo rather than monocular lies in the absolute scale that can be directly provided, while the performance of stereo VO highly depends on the ratio between the stereo baseline and the environmental depth. For high-altitude flights, stereo generally degenerates to monocular, making it ineffective for new feature depth generation. To explore this problem, we discussed a long-range stereo VO technique. On the basis of the current baseline-depth ratio, the odometry switches the working mode between short range and long range. For long range, the stereo almost degenerates to monocular, but the stereo “weak static baseline” can still provide useful physical scale information both for new map point generation and VO calculation. The new feature depth is estimated by introducing additional stereo observations through time. The performance of long-range VO was evaluated using both EuRoC datasets and our own dataset, results showing that the proposed VO improves the performance for long-range environments.

Secondly, a new state estimation system is derived to fuse both absolute state measurement sensors (GPS, barometer) and the relative 6 D.O.F pose state measurement provided by long-range VO. The EKF estimator and long-range visual estimation help each other to improve the robustness of the method. The IMU integral prediction from the EKF estimator is used both for guiding image-feature matching and long-range VO optimization. Additionally, the VO is utilized as the relative measurements for the update of the EKF state, especially for the GPS outage situations. To our best knowledge, the proposed system is the first EKF estimator for fusing both relative measurements (VO, IMU) and absolute measurements (GPS, barometer) with different time stamps. The performance of the proposed EKF estimator is investigated and compared. Results verified the effectiveness of the state estimation system.

## Figures and Tables

**Table 1:** Comparisons of path RMSE (m).

Method	RSME x	RMSE y	RMSE z
Long-range VO	1.4936	3.0465	2.2860
ORBSLAM 2.0 VO	5.0012	21.1514	3.3277
Long-range VO	5.8547	7.6728	4.5409

**Table 2:** Comparisons of path RMSE (m) for GPS outage.

Method	RSME x	RMSE y	RMSE z
EKF (300 s to 360 s, GPS lost)	1.3782	2.2670	0.5859
EKF without VO (300 s to 360 s, GPS lost)	19.8595	66.4899	0.6047
EKF (500 s to 720 s, GPS lost)	3.5654	3.8767	0.5535
EKF without VO (500 s to 720 s, GPS lost)	595.9539	141.5476	0.5973

## Supplementary Materials

The experimental video for EKF fusion estimation is available online at https://www.youtube.com/watch?v=LYszVoEboWY.
